# (*Z*)-3-(Benzyl­carbamo­yl)prop-2-enoic acid

**DOI:** 10.1107/S160053681100609X

**Published:** 2011-02-23

**Authors:** Su-Lan Dong, Xiao-Chun Cheng

**Affiliations:** a Huaiyin Institute of Technology, Huaiyin 223003, Jiangsu, People’s Republic of China; b College of Life Science and Chemical Engineering, Huaiyin Institute of Technology, Huaian, 223003, People’s Republic of China

## Abstract

The title compound, C_11_H_11_NO_3_, was synthesized by the reaction of maleic andydride and phenyl­methanamine. The mol­ecular conformation is stabilized by by an intra­molecular O—H⋯O hydrogen bond. In the crystal, mol­ecules are linked by inter­molecular N—H⋯O and C—H⋯O hydrogen bonds, forming a chain along the *b* axis.

## Related literature

For related structures, see Gowda *et al.* (2009*a* ▶,*b* ▶,*c* ▶); Prasad *et al.* (2002 ▶).
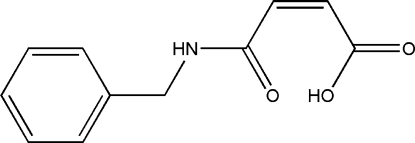


## Experimental

### 

#### Crystal data

C_11_H_11_NO_3_
*M* *_r_* = 205.21
Monoclinic, 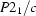

*a* = 10.651 (2) Å
*b* = 12.601 (3) Å
*c* = 8.3130 (17) Å
β = 108.44 (3)°
*V* = 1058.4 (4) Å^3^
*Z* = 4
Mo *K*α radiation
μ = 0.10 mm^−1^
*T* = 298 K
0.30 × 0.20 × 0.10 mm

#### Data collection

Enraf–Nonius CAD-4 diffractometer
Absorption correction: ψ scan (North *et al.*, 1968 ▶) *T*_min_ = 0.972, *T*_max_ = 0.991
2018 measured reflections
1913 independent reflections
1013 reflections with *I* > 2σ(*I*)
*R*_int_ = 0.022
3 standard reflections every 200 reflections  intensity decay: 1%

#### Refinement

*R*[*F* ^2^ > 2σ(*F* ^2^)] = 0.057
*wR*(*F* ^2^) = 0.175
*S* = 1.00
1913 reflections
137 parameters
H-atom parameters constrained
Δρ_max_ = 0.17 e Å^−3^
Δρ_min_ = −0.16 e Å^−3^

### 

Data collection: *CAD-4 Software* (Enraf–Nonius, 1989 ▶); cell refinement: *CAD-4 Software*; data reduction: *XCAD4* (Harms & Wocadlo, 1995 ▶); program(s) used to solve structure: *SHELXS97* (Sheldrick, 2008 ▶); program(s) used to refine structure: *SHELXL97* (Sheldrick, 2008 ▶); molecular graphics: *ORTEP-3 for Windows* (Farrugia, 1997 ▶); software used to prepare material for publication: *SHELXL97*.

## Supplementary Material

Crystal structure: contains datablocks I, global. DOI: 10.1107/S160053681100609X/kj2163sup1.cif

Structure factors: contains datablocks I. DOI: 10.1107/S160053681100609X/kj2163Isup2.hkl

Additional supplementary materials:  crystallographic information; 3D view; checkCIF report

## Figures and Tables

**Table 1:** Hydrogen-bond geometry (Å, °)

*D*—H⋯*A*	*D*—H	H⋯*A*	*D*⋯*A*	*D*—H⋯*A*
O3—H3*B*⋯O1	0.85	1.61	2.461 (3)	178
N—H0*A*⋯O2^i^	0.86	2.00	2.855 (3)	171
C9—H9*A*⋯O3^i^	0.93	2.48	3.413 (4)	177

Symmetry code: (i) 
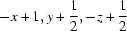
.

**Fig. 1.**
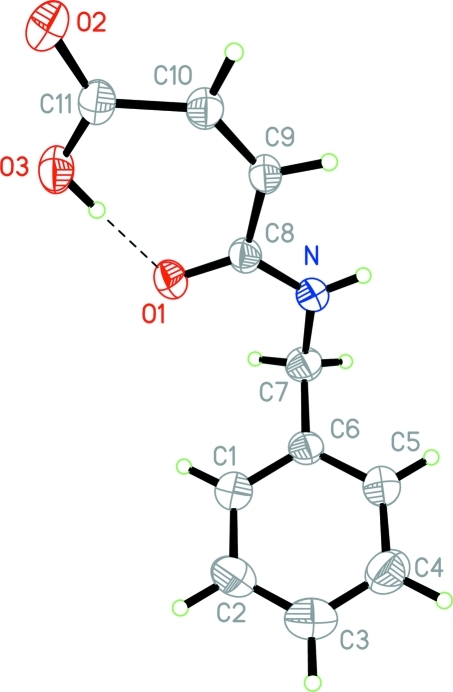
Molecular structure of the title compound. Displacement ellipsoids are drawn at the 30% probability level.

**Fig. 2.**
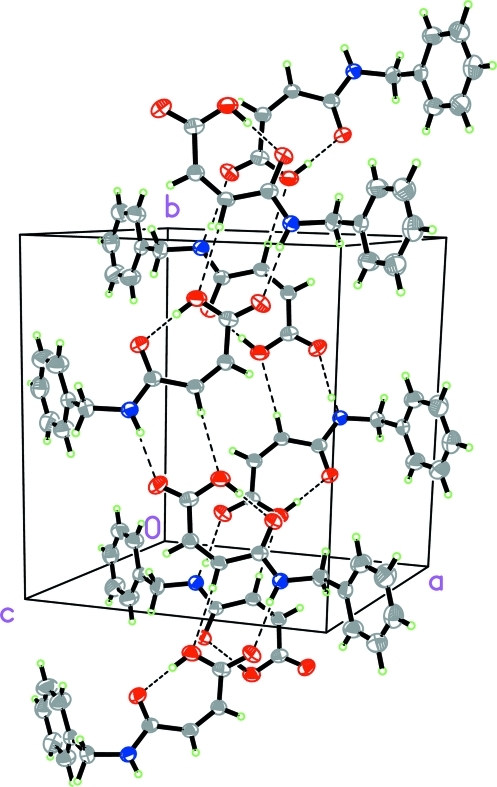
Crystal packing of the title compound. Dashed lines indicate hydrogen bonds.

## supplementary crystallographic information

### Comment

The amide moiety is an important constituent of many biologically significant compounds. As a part of studying the effect of ring and side chain substitution on the crystal structures of this class compounds (Gowda *et al.*, 2009*a*, 2009*b*, 2009*c*; Prasad *et al.*, 2002), the crystal structure of (*Z*)-4-(benzylamino)-4-oxobut-2-enoic acid has been determined. The molecular conformation (Fig. 1) is stabilized by intramolecular O–H···O bonds. As can be seen from the packing diagram (Fig.2), molecules are linked by intermolecular N–H···O and C–H···O hydrogen bonds to form a chain along the *b* axis in which they may be effective in the stabilization of structure (Table 1).

### Experimental

A solution of maleic andydride (10 g, 0.1 mol) in dichloromethane (50 ml) was added dropwise to an ice-cold solution of phenylmethanamine (10.7 g,0.1 mol) in dichloromethane (50 ml). After the addition was complete (1.5 h), the resulting suspension was stirred at ambient temperature for 20 h. A white solid was collected and washed twice with ether to give the crude product. This crude soild was partitioned between a saturated NaHCO_3_ solution and ether. The aqueous fraction was brought to pH = 1–2 with 5 N HCl in an ice bath then extracted with a (1:l) EtOAc-THF mixture. The combined organic layers were dried with Na_2_SO_4_, filtered and concentrated to give (*Z*)-4-(benzylamino)-4-oxobut-2-enoic acid as a white solid. The product was purified by repeated crystallization from methanol. Crystals of the title compound, suitable for X-ray diffraction, were obtained by slow evaporation from a solution in methanol.

### Refinement

H atoms were positioned geometrically and H-atom parameters were constrained, with O—H = 0.85 Å(for OH), N—H = 0.86 Å(for NH) and C—H = 0.93,0.93 and 0.97Å for aromatic, methylene and doublebond H, respectively, and constrained to ride on their parent atoms, with *U*_iso_(H) = *xU*_eq_(C,*N*,*O*), where *x* = 1.5 for OH, and *x* = 1.2 for all other H atoms.

### Crystal data

**Table:**

C_11_H_11_NO_3_	*F*(000) = 432
*M**_r_* = 205.21	*D*_x_ = 1.288 Mg m^−^^3^
Monoclinic, *P*2_1_/*c*	Mo *K*α radiation, λ = 0.71073 Å
Hall symbol: -P 2ybc	Cell parameters from 25 reflections
*a* = 10.651 (2) Å	θ = 9–12°
*b* = 12.601 (3) Å	µ = 0.10 mm^−^^1^
*c* = 8.3130 (17) Å	*T* = 298 K
β = 108.44 (3)°	Block, colorless
*V* = 1058.4 (4) Å^3^	0.30 × 0.20 × 0.10 mm
*Z* = 4	

### Data collection

**Table:**

Enraf–Nonius CAD-4 diffractometer	1013 reflections with *I* > 2σ(*I*)
Radiation source: fine-focus sealed tube	*R*_int_ = 0.022
graphite	θ_max_ = 25.3°, θ_min_ = 2.0°
ω/2θ scans	*h* = −12→0
Absorption correction: ψ scan (North *et al.*, 1968)	*k* = 0→15
*T*_min_ = 0.972, *T*_max_ = 0.991	*l* = −9→9
2018 measured reflections	3 standard reflections every 200 reflections
1913 independent reflections	intensity decay: 1%

### Refinement

**Table:**

Refinement on *F*^2^	Secondary atom site location: difference Fourier map
Least-squares matrix: full	Hydrogen site location: inferred from neighbouring sites
*R*[*F*^2^ > 2σ(*F*^2^)] = 0.057	H-atom parameters constrained
*wR*(*F*^2^) = 0.175	*w* = 1/[σ^2^(*F*_o_^2^) + (0.078*P*)^2^] where *P* = (*F*_o_^2^ + 2*F*_c_^2^)/3
*S* = 1.00	(Δ/σ)_max_ < 0.001
1913 reflections	Δρ_max_ = 0.17 e Å^−^^3^
137 parameters	Δρ_min_ = −0.16 e Å^−^^3^
0 restraints	Extinction correction: *SHELXL97* (Sheldrick, 2008), Fc^*^=kFc[1+0.001xFc^2^λ^3^/sin(2θ)]^-1/4^
Primary atom site location: structure-invariant direct methods	Extinction coefficient: 0.030 (5)

### Special details

**Table:**

Geometry. All e.s.d.'s (except the e.s.d. in the dihedral angle between two l.s. planes) are estimated using the full covariance matrix. The cell e.s.d.'s are taken into account individually in the estimation of e.s.d.'s in distances, angles and torsion angles; correlations between e.s.d.'s in cell parameters are only used when they are defined by crystal symmetry. An approximate (isotropic) treatment of cell e.s.d.'s is used for estimating e.s.d.'s involving l.s. planes.
Refinement. Refinement of *F*^2^ against ALL reflections. The weighted *R*-factor *wR* and goodness of fit *S* are based on *F*^2^, conventional *R*-factors *R* are based on *F*, with *F* set to zero for negative *F*^2^. The threshold expression of *F*^2^ > σ(*F*^2^) is used only for calculating *R*-factors(gt) *etc*. and is not relevant to the choice of reflections for refinement. *R*-factors based on *F*^2^ are statistically about twice as large as those based on *F*, and *R*- factors based on ALL data will be even larger.

### Fractional atomic coordinates and isotropic or equivalent isotropic displacement parameters (Å^2^)

**Table:**

	*x*	*y*	*z*	*U*_iso_*/*U*_eq_	
N	0.6963 (2)	0.4593 (2)	0.1087 (3)	0.0537 (7)	
H0A	0.6678	0.5211	0.1242	0.064*	
O1	0.6741 (2)	0.28274 (17)	0.1283 (3)	0.0636 (7)	
C1	1.0039 (4)	0.3602 (3)	0.2253 (6)	0.0904 (14)	
H1A	0.9678	0.2962	0.1767	0.108*	
O2	0.3695 (2)	0.17148 (18)	0.3280 (3)	0.0750 (8)	
C2	1.1254 (4)	0.3611 (4)	0.3536 (7)	0.1015 (16)	
H2A	1.1693	0.2975	0.3903	0.122*	
O3	0.5270 (2)	0.15868 (18)	0.2112 (3)	0.0669 (7)	
H3B	0.5786	0.2005	0.1821	0.100*	
C3	1.1799 (4)	0.4531 (4)	0.4250 (6)	0.0853 (13)	
H3A	1.2611	0.4532	0.5106	0.102*	
C4	1.1149 (4)	0.5457 (4)	0.3707 (6)	0.0871 (13)	
H4A	1.1519	0.6097	0.4188	0.104*	
C5	0.9942 (4)	0.5450 (3)	0.2443 (5)	0.0754 (11)	
H5A	0.9505	0.6088	0.2085	0.090*	
C6	0.9377 (3)	0.4525 (3)	0.1707 (4)	0.0547 (9)	
C7	0.8043 (3)	0.4522 (3)	0.0365 (4)	0.0621 (10)	
H7A	0.7989	0.5117	−0.0394	0.074*	
H7B	0.7946	0.3876	−0.0296	0.074*	
C8	0.6403 (3)	0.3750 (2)	0.1514 (4)	0.0491 (8)	
C9	0.5356 (3)	0.3986 (3)	0.2282 (4)	0.0500 (8)	
H9A	0.5219	0.4702	0.2440	0.060*	
C10	0.4586 (3)	0.3320 (3)	0.2777 (4)	0.0522 (8)	
H10A	0.3995	0.3648	0.3235	0.063*	
C11	0.4498 (4)	0.2143 (3)	0.2731 (4)	0.0556 (9)	

### Atomic displacement parameters (Å^2^)

**Table:**

	*U*^11^	*U*^22^	*U*^33^	*U*^12^	*U*^13^	*U*^23^
N	0.0488 (16)	0.0458 (15)	0.0658 (18)	0.0025 (13)	0.0169 (14)	0.0026 (13)
O1	0.0654 (15)	0.0463 (13)	0.0842 (17)	0.0078 (12)	0.0306 (13)	−0.0028 (12)
C1	0.064 (2)	0.062 (3)	0.134 (4)	0.000 (2)	0.016 (3)	−0.001 (2)
O2	0.0792 (17)	0.0585 (15)	0.094 (2)	−0.0142 (14)	0.0373 (16)	0.0101 (14)
C2	0.068 (3)	0.083 (3)	0.141 (4)	0.014 (2)	0.014 (3)	0.025 (3)
O3	0.0856 (18)	0.0436 (13)	0.0764 (16)	−0.0009 (12)	0.0326 (15)	0.0007 (12)
C3	0.066 (3)	0.109 (4)	0.078 (3)	0.001 (3)	0.020 (2)	−0.001 (3)
C4	0.077 (3)	0.086 (3)	0.097 (3)	−0.011 (3)	0.025 (3)	−0.027 (3)
C5	0.070 (2)	0.061 (3)	0.088 (3)	0.002 (2)	0.014 (2)	−0.003 (2)
C6	0.0485 (19)	0.055 (2)	0.065 (2)	−0.0001 (17)	0.0250 (17)	0.0034 (18)
C7	0.059 (2)	0.066 (2)	0.067 (2)	−0.0028 (18)	0.0293 (19)	0.0036 (18)
C8	0.0486 (19)	0.0436 (18)	0.0496 (19)	0.0012 (16)	0.0075 (15)	−0.0011 (15)
C9	0.055 (2)	0.0375 (17)	0.057 (2)	0.0008 (15)	0.0162 (17)	−0.0012 (15)
C10	0.058 (2)	0.0462 (18)	0.054 (2)	0.0006 (17)	0.0205 (17)	0.0011 (16)
C11	0.061 (2)	0.0463 (19)	0.053 (2)	−0.0039 (19)	0.0084 (17)	0.0024 (17)

### Geometric parameters (Å, °)

**Table:**

N—C8	1.320 (4)	C3—H3A	0.9300
N—C7	1.459 (4)	C4—C5	1.379 (5)
N—H0A	0.8600	C4—H4A	0.9300
O1—C8	1.250 (3)	C5—C6	1.365 (5)
C1—C6	1.361 (5)	C5—H5A	0.9300
C1—C2	1.392 (6)	C6—C7	1.503 (4)
C1—H1A	0.9300	C7—H7A	0.9700
O2—C11	1.216 (4)	C7—H7B	0.9700
C2—C3	1.347 (6)	C8—C9	1.480 (4)
C2—H2A	0.9300	C9—C10	1.327 (4)
O3—C11	1.304 (4)	C9—H9A	0.9300
O3—H3B	0.8501	C10—C11	1.485 (4)
C3—C4	1.360 (6)	C10—H10A	0.9300
			
C8—N—C7	122.9 (3)	C1—C6—C7	121.0 (3)
C8—N—H0A	118.5	C5—C6—C7	120.9 (3)
C7—N—H0A	118.5	N—C7—C6	112.2 (3)
C6—C1—C2	120.5 (4)	N—C7—H7A	109.2
C6—C1—H1A	119.8	C6—C7—H7A	109.2
C2—C1—H1A	119.8	N—C7—H7B	109.2
C3—C2—C1	120.7 (4)	C6—C7—H7B	109.2
C3—C2—H2A	119.6	H7A—C7—H7B	107.9
C1—C2—H2A	119.6	O1—C8—N	122.1 (3)
C11—O3—H3B	108.9	O1—C8—C9	123.1 (3)
C2—C3—C4	119.3 (4)	N—C8—C9	114.8 (3)
C2—C3—H3A	120.4	C10—C9—C8	129.1 (3)
C4—C3—H3A	120.4	C10—C9—H9A	115.5
C3—C4—C5	120.1 (4)	C8—C9—H9A	115.5
C3—C4—H4A	120.0	C9—C10—C11	131.6 (3)
C5—C4—H4A	120.0	C9—C10—H10A	114.2
C6—C5—C4	121.3 (4)	C11—C10—H10A	114.2
C6—C5—H5A	119.3	O2—C11—O3	121.0 (3)
C4—C5—H5A	119.3	O2—C11—C10	118.7 (3)
C1—C6—C5	118.1 (4)	O3—C11—C10	120.3 (3)
			
C6—C1—C2—C3	−0.4 (7)	C1—C6—C7—N	98.2 (4)
C1—C2—C3—C4	0.1 (7)	C5—C6—C7—N	−80.0 (4)
C2—C3—C4—C5	0.3 (7)	C7—N—C8—O1	−1.9 (5)
C3—C4—C5—C6	−0.3 (6)	C7—N—C8—C9	177.9 (3)
C2—C1—C6—C5	0.4 (6)	O1—C8—C9—C10	−3.0 (5)
C2—C1—C6—C7	−177.9 (4)	N—C8—C9—C10	177.2 (3)
C4—C5—C6—C1	−0.1 (6)	C8—C9—C10—C11	−0.3 (5)
C4—C5—C6—C7	178.2 (4)	C9—C10—C11—O2	179.7 (3)
C8—N—C7—C6	−89.9 (4)	C9—C10—C11—O3	−0.3 (5)

### Hydrogen-bond geometry (Å, °)

**Table:**

*D*—H···*A*	*D*—H	H···*A*	*D*···*A*	*D*—H···*A*
O3—H3B···O1	0.85	1.61	2.461 (3)	178
N—H0A···O2^i^	0.86	2.00	2.855 (3)	171
C9—H9A···O3^i^	0.93	2.48	3.413 (4)	177

Symmetry codes: (i) −*x*+1, *y*+1/2, −*z*+1/2.
